# Work disability and its determinants in patients with pituitary tumor-related disease

**DOI:** 10.1007/s11102-018-0913-3

**Published:** 2018-10-04

**Authors:** Daniel J. Lobatto, Anath N. V. Steffens, Amir H. Zamanipoor Najafabadi, Cornelie D. Andela, Alberto M. Pereira, Wilbert B. van den Hout, Wilco C. Peul, Thea P. M. Vliet Vlieland, Nienke R. Biermasz, Wouter R. van Furth

**Affiliations:** 1 Department of Neurosurgery, Leiden University Medical Center, Albinusdreef 2, 2333 ZA Leiden, The Netherlands; 2 Division of Endocrinology and Center for Endocrine Tumors, Department of Medicine, Leiden University Medical Center, Leiden, The Netherlands; 3 Medical Decision Making, Department of Biomedical Data Sciences, Leiden University Medical Center, Leiden, The Netherlands; 4 Department of Neurosurgery, Haaglanden Medical Center, The Hague, The Netherlands; 5 Department of Orthopedic Surgery, Leiden University Medical Center, Leiden, The Netherlands

**Keywords:** Pituitary adenoma, Work disability, Employment, Absenteeism, Presenteeism, Health-related quality of life

## Abstract

**Introduction:**

Pituitary tumors may have a considerable impact on patients’ functional status, including paid employment, yet research in this area is sparse.

**Objective:**

To describe work disability and its determinants in patients treated for a pituitary tumor.

**Methods:**

Cross-sectional study including patients treated for a pituitary tumor in the working age (18–65 years), who completed five validated questionnaires assessing work disability [Short Form-Health and Labour Questionnaire, Work Role Functioning Questionnaire 2.0 (WRFQ)], health-related quality of life (HRQoL) and utility (Short Form-36, EuroQoL) and disease burden (Leiden Bother and Needs Questionnaire-Pituitary). Additional data were extracted from the medical records (age, gender, tumor type, treatment, date of diagnosis) and self-reports (marital status, education, endocrine status). Associations of disease-specific and sociodemographic characteristics, HRQoL, and disease burden with (not) having a paid job were examined through multivariate logistic regression.

**Results:**

We included 241 patients (61% female, median age 53 years, median time since diagnosis 11 years), of whom 68 (28%) were without a paid job. Patients who had acromegaly, Cushing’s disease, (pan)hypopituitarism, radiotherapy, were single, less educated, lower HRQoL, and increased disease burden were more often without a paid job (p < 0.05). Among those with paid jobs, 41% reported health-related absenteeism in the previous year. The three work incapacitating problems reported by the largest proportion of patients were within the mental and social domain (WRFQ).

**Conclusion:**

Work disability among patients treated for a pituitary tumor is substantial. As impact on social functioning is high, it is strongly advised to incorporate work disability during clinical guidance of patients.

**Electronic supplementary material:**

The online version of this article (10.1007/s11102-018-0913-3) contains supplementary material, which is available to authorized users.

## Introduction

Even long after multimodality treatment of pituitary tumors, many patients report impairments in health-related quality of life (HRQoL) [1–3]. An important, but relatively underinvestigated domain of HRQoL is the impact of pituitary tumors on societal participation, with special regard to having or maintaining a paid job.

Our recent focus group study in patients addressing the patient’s perspective on disease burden and needs for support indicated that chronic pituitary conditions have a significant impact on work and financial status [4]. In that qualitative study, several patients expressed experiencing a lack of understanding by employers, medical specialists, and occupational physicians. Some patients feared losing their jobs and therefore refrained from informing their employers/co-workers about their disease or from mentioning it during job interviews. Furthermore, a number of patients reported not being able to continue their jobs because they could not perform the same tasks they used to do [4].

Quantitative studies on the extent of work disability in terms of having a paid job or not in this disease area are scarce. Rates of patients without a paid job vary between 36 and 74% [5–9]; however, these studies report a variety of pituitary conditions [7–9], and/or do not exclusively comprise of patients of working age [5, 6, 8, 9], therefore affecting these rates. Determinants of having a paid job or not have not been studied extensively.

Work disability, however, not only comprises having paid employment or not; sick leave (absenteeism) or not being productive while at work (presenteeism, in some countries defined as hidden absenteeism) are also important aspects. Previous studies have reported absenteeism rates varying between 19.8 and 40.2 days per year [5, 10–12], while presenteeism and perceived problems at work have never been addressed in patients with pituitary tumors. More insight into the perceived problems may help patients and healthcare providers, including occupational physicians, in guiding patients.

The objectives of this current study were to investigate (1) the rates of patients with and without a paid job treated for a pituitary tumor and of working age; (2) determinants of not having a paid job in this group; (3) loss of productivity in patients with a paid job defined as absenteeism and presenteeism; and (4) patients’ perceived problems at work.

## Patients and methods

### Study design

This cross-sectional survey study among a cohort of patients treated for a pituitary tumor in a chronic care setting was performed between September 2016 and March 2017 at a tertiary referral center, the Leiden University Medical Center (LUMC) in the Netherlands. Institutional medical ethical review board approval was obtained prior to the start of the study (p12.067).

### Study population

Patients included in this study were part of a larger cross-sectional cohort (N = 400) on long-term outcomes among patients treated for a pituitary tumor, including all patients diagnosed with a pituitary tumor (non-functioning pituitary adenoma (NFPA), acromegaly (ACRO), Cushing’s disease (CD), prolactinoma (PRL) or Rathke’s cleft cyst (RCC)), with sufficient Dutch language skills, more than 6 months of treatment, and currently under active follow-up. A subset of this study, patients between the ages of 18 and 65, was eligible for the present study. Patients were identified through the hospital registries. Diagnosis was confirmed prior to invitation by means of review of the medical record by DJL. Eligible patients were invited by a written letter to participate in this study; after written informed consent was obtained, a questionnaire was sent to all participants. In case of non-response, patients were re-approached by regular mail or by telephone.

### Assessments

A set of 5 validated questionnaires was administered, including measures on work status and productivity [Short-Form-Health and Labour Questionnaire (SF-HLQ)], work-related difficulties [work role functioning questionnaire 2.0 (WRFQ)], HRQoL and utility [Short Form-36 (SF-36) and EuroQoL (EQ-5D)], and perceived bother and needs for support [Leiden Bother and Needs for Support Questionnaire for pituitary patients (LBNQ-Pituitary)], and questions on current medication usage, as well as visits to an occupational physician. The questionnaires could be filled in either digitally or on paper, both shown to provide equivalent results [13].

### Disease-specific and sociodemographic characteristics

The disease-specific and sociodemographic characteristics were collected from the digital medical records (tumor type, date of diagnosis) and self-reports (educational level, marital status, endocrine status). This included age, sex, and hormonal status (with hypopituitarism defined as replacement of ≥ 1 endocrine deficits, and panhypopituitarism as ≥ 2 plus hydrocortisone replacement). Treatment was categorized arbitrarily into 4 categories: (1) no treatment (including patients with discontinued medication), (2) ongoing medical (tumor) treatment (including those with prior surgery and/or radiotherapy), (3) postoperative patients (excluding ongoing medical treatment and irradiated patients), (4) a history of radiotherapy (including prior surgery and/or discontinued medication). The categorization among patients with multimodality treatment was done according to the supposed largest ongoing impact in the current chronic setting: (1) ongoing medication (greatest impact), (2) radiotherapy, (3) past surgical intervention, (4) no therapy or temporary medical treatment (least impact). A detailed description of the treatment algorithm, which was in line with existing guidelines, has previously been published [14–16]. Level of education was categorized into low, intermediate or high, based on the guidelines of Statistics Netherlands (CBS) [17], which correspond with the International Standard Classification of Education Fields of Training and Education 2013 of the UNESCO [18].

### Work status

Work status was assessed through two questionnaires: a selection of the *SF-HLQ* was used to obtain information about employment, absenteeism and presenteeism over the past 12 months. Employment was divided into two categories: (1) having a paid job, which included part-time paid jobs, some combined with other duties such as education, taking care of the household or receiving a work disability pension; (2) not having a paid job, including engagement in any non-paid duties, education, taking care of the household, voluntary and involuntary unemployment, or early pension. The questionnaire did not differentiate whether voluntary and involuntary unemployment/early pension was due to the illness or due to other reasons. Absenteeism was defined as the number of days on sick leave over the past 12 months, presenteeism as reduced productivity while at work, and assessed by means of a question on the self-perceived performance at work on a scale of 1–10 [19].

The *WRFQ* assesses work disabilities and consists of 27 questions, divided into four subcategories including: work scheduling and output demands (WSOD), physical demands (PD), mental & social demands (MSD), and flexibility demands (FD) from which an index score was calculated. Items were scored on a 5-point rating scale: (1) all the time (100%), (2) most of the time, (3) half of the time (50%), (4) some of the time, (5) completely not (0%). All items included the option “not applicable to my job” [20]. Higher scores indicate less self-perceived work disabilities.

### Health-related quality of life and utility

The *SF-36* is a 36-item questionnaire which covers eight domains: physical function, physical role, bodily pain, general health, vitality, social function, emotional role, and mental health. These subscales range from 0 to 100, from which the physical and mental component score can be calculated. Higher scores indicate better HRQoL [21].

The *EQ-5D* (5-level) consists of 5 domains: mobility, self-care, usual activities, pain/discomfort, and anxiety/depression, from which utility (range 0–1) can be calculated (EQ-5D index). The EQ-5D also includes a visual analog scale (VAS), which records self-reported health status (range 0–100). Higher scores indicate a better perceived health status [22].

### Perceived bother and needs for support

The LBNQ-Pituitary is a disease-specific questionnaire, which was developed through focus group interviews with patients [23]. For this study, the LBNQ-Pituitary consisted of 26 items divided into five subscales: mood problems, negative illness perceptions, issues in sexual functioning, physical and cognitive complaints, and issues in social functioning, from which an index score can be calculated (range 0–100). A detailed description of how the items are scored has been previously published. Higher scores indicate a greater bother [23]. For this study we added a question on the usage of and number of visits to an occupational physician.

### Statistical analysis

Data entry and control was performed through an online survey platform. All statistical analyses were performed with SPSS 23.0 software (IBM SPSS Inc., Armonk, USA). Numerical variables are presented as means and standard deviations (SD) or medians with interquartile ranges (IQR), nominal variables as frequencies with percentages.

For the univariate analysis, a Chi square test was performed for categorical variables, student’s *T* test or Mann–Whitney *U* tests for numerical variables where applicable. Logistic regression analysis was used to determine the relationship between work status (paid job/no paid job) as a dependent factor and all possible contributing factors (i.e., disease-specific characteristics, sociodemographic characteristics, HRQoL). To control for confounding, variables associated with both the determinant and the outcome and not in the causal pathway of the relationship of interest were used as covariates in the multivariate analysis [24]. All determinants were corrected for age and gender, depending on the determinant also for tumor type, treatment and/or QoL. For the work disability analysis, variables were compared between tumor types via AN(C)OVA, corrected for age and gender where applicable. For all analyses, the level of significance was set at p < 0.05 (two-sided) and associations are expressed as odds ratios (ORs) with the corresponding 95% confidence interval (CI). Missing data on the questionnaires was handled by complete case analysis due to the low amount of missings (< 5%).

## Results

### Study population (Fig. 1)

**Fig. 1:**
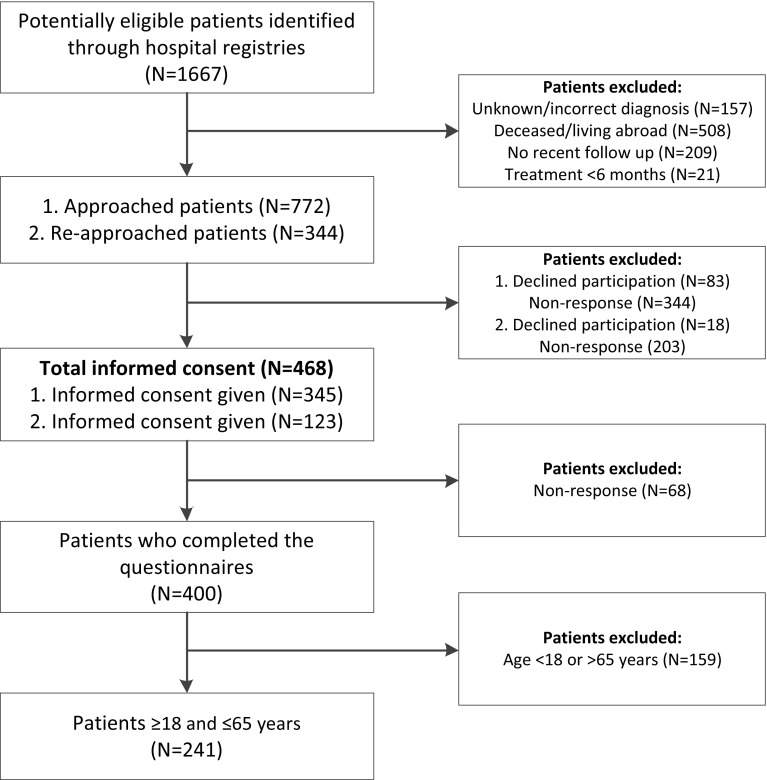
Flow chart of in-/exclusion of patients with a pituitary tumor

A total of 1667 patients were identified from the hospital registry. After exclusion of ineligible patients, letters were sent to 772 patients (including patients > 65 years), enrolling a total of 400 (51.8%) patients of whom 241 (60.2%) patients between 18 and 65 years of age.

### Patient characteristics (Table 1)

**Table 1:** Characteristics of 241 patients with a pituitary tumor and of working age and comparisons between those working and not working

	Total (N = 241)	No paid job (N = 68)	Paid job (N = 173)	P-value
Sociodemographic characteristics				
Female gender, N (%)	147 (61.0)	44 (32.0)	103 (68.0)	0.459
Age in years, median (IQR)	53.0 (45.0–59.5)	54.7 (45.6–60.7)	52.4 (44.9–59.2)	0.114
Marital status, N (%)				
Relationship/married	190 (79.2)	48 (70.6)	142 (82.6)	**0.040**
Education, N (%)				
Low	65 (27.0)	27 (39.7)	38 (22.0)	< **0.001**
Intermediate	60 (24.9)	22 (32.4)	38 (22.0)	
High	115 (47.7)	19 (27.9)	96 (55.5)	
Disease-specific characteristics				
Tumor type, N (%), % per tumor type				
NFPA	65 (27.0)	15 (23.1)	50 (76.9)	**0.021**
ACRO	41 (17.0)	16 (39.0)	25 (61.0)	
CD	32 (13.3)	15 (46.9)	17 (53.1)	
PRL	97 (40.2)	20 (20.6)	77 (79.4)	
RCC	6 (2.5)	2 (33.3)	4 (66.7)	
Time since diagnosis in years, median (IQR)	11.4 (5.2–20.5)	10.8 (5.1–19.0)	13.7 (6.6–23.8)	0.204
Treatment, N (%)				
No treatment/discontinued medication	36 (14.9)	6 (8.8)	30 (17.3)	**0.022**
Ongoing medication	75 (31.1)	17 (25.0)	58 (33.5)	
Surgery	96 (39.8)	29 (42.6)	67 (38.7)	
Radiotherapy	34 (14.1)	16 (23.5)	18 (10.4)	
Endocrine status, N (%)				
No deficits	112 (46.5)	18 (26.5)	94 (54.3)	< **0.001**
Hypopituitarism	84 (34.9)	34 (50.0)	50 (28.9)	
Panhypopituitarism	45 (18.7)	16 (23.5)	29 (16.8)	
HRQoL and disease bother				
EQ-5D score, mean (SD)^a^	0.908 (0.08)	0.862 (0.12)	0.926 (0.06)	< **0.001**
EQ-5D VAS, mean (SD)^a^	73.32 (20.7)	63.88 (19.1)	77.02 (20.1)	< **0.001**
SF-36 PCS, mean (SD)^a^	46.53 (10.5)	39.91 (11.5)	49.13 (8.9)	< **0.001**
SF-36 MCS, mean (SD)^a^	47.84 (12.0)	44.41 (13.3)	49.19 (11.1)	**0.005**
LBNQ-pituitary total score, mean (SD)^b^	17.37 (18.9)	25.99 (20.8)	13.98 (17.0)	< **0.001**

Due to rounding, not all percentages of the categorical variables add up to 100%

Bold—p < 0.05

*NFPA* non-functioning pituitary adenoma, *ACRO* acromegaly, *CD* Cushing’s disease, *PRL* prolactinoma, *RCC* Rathke’s cleft cyst, *N* number, *SD* standard deviation, *IQR* interquartile range, *EQ-5D* EuroQoL, *SF-36* short form-36, *LBNQ-Pituitary* Leiden bother and needs questionnaire-pituitary, *VAS* visual analogue scale, *MCS* mental component scale, *PCS* physical component scale

^a^Higher scores indicate better HRQoL

^b^Lower scores indicate lower disease burden

The 241 patients (61% female) included in the study had a median age of 53.0 (IQR 45.0–59.5) years. Median time since diagnosis was 11.4 (IQR 5.2–20.5) years and almost half of the patients were highly educated (48%). Tumor type was: (1) NFPA in 65 patients (27%), (2) ACRO in 41 patients (17%), (3) CD in 32 patients (13%), (4) PRL in 97 patients (40%), and (5) RCC in 6 patients (3%). Many patients had undergone multimodality treatment, with most patients in the surgical group (40%), followed by ongoing medical therapy (31%). (Pan)hypopituitarism was present in 129 (54%) of patients.

### Work status (Table 1)

Sixty-eight (28%) patients did not have a paid job. This proportion was highest in patients with Cushing’s disease (15/32, 47%) and lowest in patients with a prolactinoma (20/97, 21%). Those without a job did not differ with respect to age [median age of 54.7 (45.6–60.7) years] compared to those with a job [median age of 52.4 (44.9–59.2) years], however, there was a tendency to a lower education level (40% vs. 22% low education, intermediate 32% vs. 22%), and more endocrine deficits (74% vs. 46%) (Table 1). The following reasons were reported for not having a paid job: 1) being a scholar/student (3%), taking care of the household (31%), receiving an early pension (9%), having a (partial) disability pension (41%) or another reason [i.e. involuntary unemployment or performing charity work (16%)] (Supplementary Fig. 1). Of those with a (partial) disability pension, 21 out of 28 received full-disability pensions (80–100% disability).

### Determinants for having a paid job or not (Table 2)

**Table 2:** Univariate and multivariate analysis of determinants for not having a paid job among pituitary tumors of working age

Determinant	Crude			Adjusted for disease-specific and sociodemographic characteristics			Adjusted for disease-specific, sociodemographic characteristics and HRQoL		
	OR	95% CI	p-value	OR	95% CI	p-value	OR	95% CI	p-value
Female gender	1.25	0.70–2.23	0.460	–	–	–	–	–	–
Age	1.02	0.99–1.05	0.218	–	–	–	–	–	–
Marital status (ref: relationship/married)^a^									
Single/divorced/widow	**1.97**	**1.03–3.79**	**0.042**	**2.27**	**1.15–4.49**	**0.019**	1.77	0.80–3.87	0.157
Education (ref: high)^a^									
Intermediate	**3.59**	**1.79–7.21**	< **0.001**	**3.43**	**1.70–6.93**	**0.001**	2.13	0.96–4.75	0.064
Low	**2.93**	**1.42–6.01**	**0.003**	**2.99**	**1.45–6.17**	**0.003**	**3.31**	**1.48–7.42**	**0.004**
Tumor type (ref: PRL)^a^									
NFPA	1.16	0.54–2.47	0.710	1.11	0.50–2.44	0.803	0.84	0.35–2.04	0.698
ACRO	**2.46**	**1.11–5.47**	**0.027**	**2.73**	**1.19–6.29**	**0.018**	1.96	0.77–4.99	0.161
CD	**3.40**	**1.45–8.00**	**0.005**	**3.25**	**1.38–7.67**	**0.007**	**2.86**	**1.10–7.45**	**0.032**
RCC	1.93	0.33–11.27	0.468	1.68	0.28–9.97	0.566	0.97	0.13–7.34	0.979
Time since diagnosis^b^	1.02	0.99–1.05	0.154	1.00	0.96–1.03	0.860	1.02	0.98–1.05	0.375
Treatment (ref: no treatment)^c^									
Ongoing medication	1.47	0.52–4.10	0.467	1.35	0.45–4.00	0.594	1.25	0.37–4.22	0.716
Surgery	2.16	0.81–5.76	0.122	1.55	0.50–4.77	0.445	1.43	0.41–5.05	0.576
Radiotherapy	**4.44**	**1.47–13.42**	**0.008**	**3.87**	**1.11–13.45**	**0.033**	3.04	0.74–12.51	0.123
Endocrine status (ref: no deficits)^b^									
Hypopituitarism	**3.55**	**1.82–6.92**	< **0.001**	**3.76**	**1.84–7.68**	< **0.001**	**3.97**	**1.77–8.88**	**0.001**
Panhypopituitarism	**2.88**	**1.31–6.36**	**0.009**	**2.87**	**1.16–7.12**	**0.023**	**2.94**	**1.06–8.17**	**0.039**

Included for HRQoL: EQ-index, MCS, PCS, LBNQ-P index

Bold—p < 0.05

*Ref* reference category, *OR* odds ratio, *CI* confidence interval

^a^Adjusted for age, gender (HRQoL)

^b^Adjusted for age, gender, tumor type, treatment (HRQoL)

^c^Adjusted for age, gender, tumor type (HRQoL)

After correcting for relevant confounders, patients diagnosed with Cushing’s disease (range OR 2.9–3.3) or acromegaly (OR 2.5–2.7) were more often without a paid job, compared to patients with a NFPA or prolactinoma; had undergone radiotherapy more often compared to no current treatment (OR 3.9); or had one or more endocrine deficits compared to patients without any deficits (OR 2.9–3.6). Furthermore, patients not currently in a relationship were more often without a paid job (OR 2.3), as well as patients with a low or intermediate education (OR 3.0–3.4). When correcting for HRQoL, the relationship between the determinants low education, Cushing’s disease, endocrine status and having a job or not remained significant.

### Working problems among patients with a paid job (Table 3)

**Table 3:** Patient and work characteristics among 173 patients of working age with a pituitary tumor and a paid job, stratified per tumor type

	Total (N = 173)	NFPA (N = 50)	ACRO (N = 25)	CD (N = 17)	PRL (N = 77)	RCC (N = 4)	p-value
SF-HLQ							
Working hours/week, median (IQR)	36.0 (24.0–40.0)	36.0 (28.0–40.0)	40.0 (32.0–40.0)	32.0 (22.0–40.0)	32.0 (24.0–40.0)	39.0 (29.0–45.0)	0.211^b^
Bothered by health-related problems during work, N (%)	68 (39.3)	22 (44.0)	9 (36.0)	5 (29.4)	30 (39.0)	2 (50.0)	0.608^b^
Performance at work despite health-related problems, mean among those bothered (SD) (scale 1–10)^a^	6.8 (1.7)	6.3 (1.6)	7.9 (1.4)	7.6 (1.5)	6.6 (1.7)	8.5 (0.7)	**0.025** ^b^
Absence from work during the past year due to health-related problems, N (%)	70 (40.5)	25 (50.0)	7 (28.0)	8 (47.1)	27 (35.1)	3 (75.0)	0.104^b^
Days absent during previous year, median days (IQR)	5.0 (4.0–28.0)	10.0 (4.0–30.0)	10.0 (5.0–35.0)	5 (4.5–5.0)	5 (3.0–30.0)	10 (3.0–130.0)	0.424^b^
Medical consumption							
Contact with occupational physician, N (%)	21 (12.1)	8 (16.0)	3 (12.0)	1 (5.9)	8 (10.4)	1 (25.0)	0.706
WRFQ (scale 0–100)							
Work scheduling and output demands, mean (SD)^a^	78.0 (28.7)	77.0 (28.3)	81.8 (27.0)	84.1 (29.5)	76.8 (28.8)	63.3 (52.7)	0.714^b^
Physical demands, mean (SD)^a^	84.3 (27.1)	83.7 (28.6)	87.2 (25.2)	83.9 (24.1)	84.5 (26.6)	68.3 (54.8)	0.849^b^
Mental demands and social demands, mean (SD)^a^	75.6 (31.2)	85.2 (24.8)	84.0 (28.0)	76.6 (28.6)	60.7 (52.9)	77.9 (29.2)	0.380^b^
Flexibility demands, mean (SD)^a^	79.8 (29.1)	76.1 (30.3)	86.3 (25.2)	84.1 (27.9)	79.6 (29.0)	68.3 (50.6)	0.563^b^
Index score, mean (SD)^a^	78.6 (28.1)	77.2 (28.6)	83.1 (26.5)	85.7 (25.4)	77.8 (27.1)	50.4 (51.3)	0.179^b^

*NFPA* non-functioning pituitary adenoma, *ACRO* acromegaly, *CD* Cushing’s disease, *PRL* prolactinoma, *RCC* Rathke’s cleft cyst, *N* number, *SD* standard deviation, *IQR* interquartile range, *SF-HLQ* short form-health and labour questionnaire, *WRFQ* work role functioning questionnaire 2.0

Bold—p < 0.05

^a^Higher scores indicate better performance at work

^b^Corrected for age and gender

Patients with a pituitary tumor and a paid job report a median number of 36 working hours per week (IQR 26.0–40.0), which was not significantly different between various tumor types. In total, 41% of patients with a paid job reported to have missed on average 27.1 (SD 54.5) days during the previous year due to illness (absenteeism) and 39% of patients reported being bothered by health-related problems during work (range per tumor type: 29–50%). Among those bothered, there was a significant difference between tumor types regarding performance at work despite health-related problems (presenteeism: mean 6.8, range per tumor type 6.3–8.5, p = 0.03). Only 21 patients (12%) were under treatment of an occupational physician during the previous year.

**Fig. 2:**
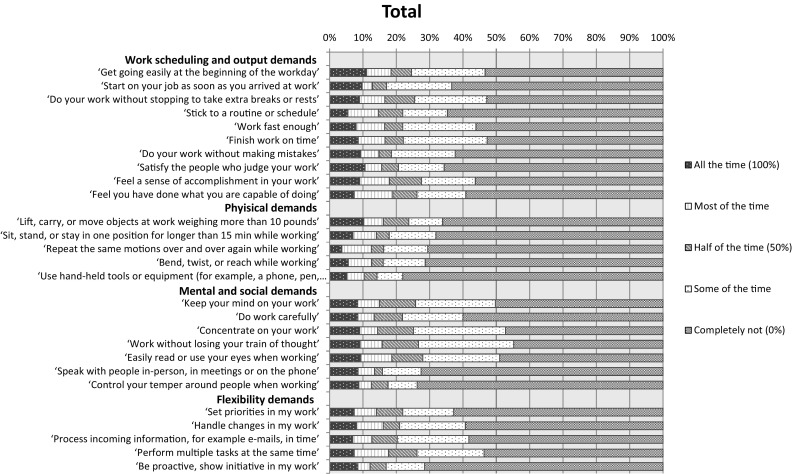
Difficulties experienced at work among patients with a paid job

The highest percentage of patients reported problems with mental and social demands (i.e. concentrating on work tasks and working without losing train of thought) (Supplementary Table 3, Fig. 2).

### Quality of life (Fig. 3)

**Fig. 3:**
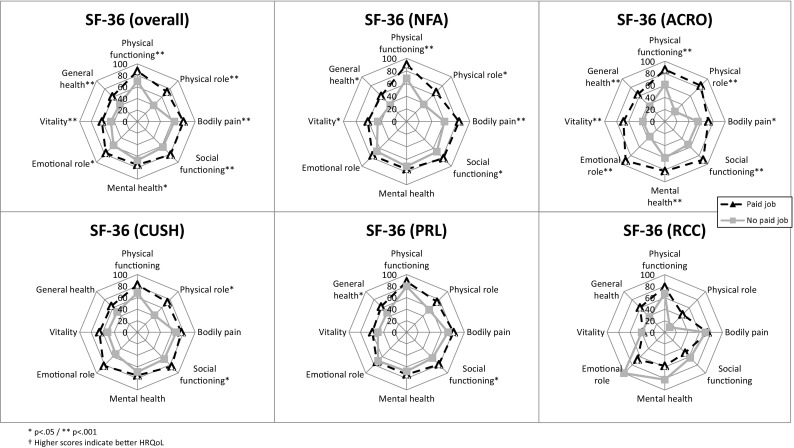
SF-36 scores in patients with a paid job compared to those without a paid job

In general, HRQoL was significantly higher among patients with a paid job compared to those without a paid job on all domains of the SF-36 (range mean difference 7.6–32.8, p < 0.05). This was confirmed among patients with acromegaly in a subgroup analysis between patients with and without a paid job, however could only be confirmed for some, but not all domains for patients with other tumor types (NFPA, CD, PRL).

## Discussion

This large cross-sectional study in patients treated for a pituitary tumor and of working age reveals that 32% of patients do not have a paid job. We found an increased risk of not having a paid job among patients with Cushing’s disease, acromegaly, (pan)hypopituitarism and/or those patients that had undergone radiotherapy. In addition, well-known general determinants such as being single or lower education are also valid in this condition. Of those with a paid job, relatively many reported having missed work due to illness during the past year (41%) or not performing up to their self-perceived maximum potential (39%). The most common problems reported by patients concerned mental and social demands of work. This study is the first to look at determinants of (not) having a paid job in patients with a pituitary tumor. Furthermore, to the best of our knowledge, this study represents the largest study to date that looks at work disability and the relationship between job status and HRQoL in this population.

### Rates of patients with a paid job

Data on work disability among patients with a history of a pituitary disease are scarce. The overall rate of 68% of patients with a paid job found in our study could not be verified in other studies, however, it is lower when compared to the general Dutch population (78.6%) (matched for age, gender and education) [25]. Two studies reported lower rates of patients with a paid job. Van Roijen et al. studied a cohort of 129 patients with hypopituitarism and found that only 26% of patients had a paid job [5]. While they did not report data within the same age range, the study also took place in 1989, further limiting comparability. Likewise, Brod et al. showed that 56% of adult patients with growth hormone deficiency had a paid job, however their results should be interpreted with caution, since they present data of a heterogeneous group of 39 patients, including patients with short stature, brain tumors, and trauma [9].

Among patients with functioning tumors, the reported rates were more comparable to ours. Wagenmakers et al. prospectively studied 123 Dutch patients in remission of CD of whom 51% had a paid job [8]. While these results are indeed in line with our findings among patients with CD (53%), the study lacked information exclusively on patients aged 18–65. Short-term outcomes, on the other hand, as found by Pikkarainen et al., were higher (66% with a paid job), however the population represented patients with Cushing’s syndrome (26 adrenal and 48 pituitary adenomas) and data was collected retrospectively, both limiting interpretability [7].

In contrast to the long-term outcomes, Jahangiri et al. looked at rates prior to diagnosis/treatment, and did not find significant differences between 18 patients with apoplexia compared to 117 patients without apoplexia [26]. These results were also collected retrospectively, had many missing data and lacked long-term results, therefore also limiting comparability of results.

### Determinants for not having a paid job

This study is the first to study determinants for not having a job in a chronic setting of patients with pituitary tumors. We found tumor type, treatment, endocrine status (disease-specific), marital status, and education level (sociodemographic) to increase the risk of not having a paid job. The disease-specific and sociodemographic determinants found in the present study are in agreement with those affecting HRQoL [1, 27–29] in pituitary and other diseases [30, 31].

In the short-term, Jahangiri et al. did not find a difference between patients with apoplexia and without apoplexia regarding having a paid job or not [6]. A history of apoplexia is probably of less importance in the chronic phase, as well as in functioning tumors.

As anticipated, patients with (pan)hypopituitarism were significantly more often without a paid job compared to those without endocrine deficits. In our analysis hypopituitarism patients performed worse than those with panhypopituitarism. The variable composition of number and severity of the number of deficiencies and replacement status limits the exact interpretation of this finding.

### Absenteeism, problems experienced at work and HRQoL

We found a relatively high percentage of patients (41%) with absence from work due to health-related reasons during the past year. Regarding the magnitude of absenteeism, our data were skewed, with the median being 5 days per year, whereas the mean was 27.1 days. The latter was considerably higher than that of the average Dutch population (8.8 days per year) (matched for age, gender) [32], however, unfortunately could not be compared to matched controls. In line with our findings, Jonsson et al. found increased sick leave in patients with NFPA (mean leave 40.2 days) compared to age-matched controls (24.0 days) [10], and other studies reported means varying between 19.8 and 38.4 days per year [5, 11, 12].

The highest percentage of patients without a paid job was among patients with ACRO/CD. The difference in HRQoL between patients with and without a paid job was largest among patients with ACRO (Fig. 3), perhaps indicating the presence of a mild and severe phenotype of patients with ACRO. Previous studies have endorsed these thoughts on various subtypes of acromegaly [33–35], however none incorporated the long-term outcomes (as depicted in Tier 3 of the value-based healthcare model [36]) on social participation and sustainability of health, such as work disability.

Prior to the study we had anticipated finding lower overall work functioning scores as measured with the WRFQ, particularly for patients with ACRO and CD. In the total cohort, we indeed found lower average scores compared to those of a large selection of Dutch employees [20], and found scores comparable to those of Dutch cancer survivors [37], which emphasizes the impact of having a pituitary tumor. Unexpectedly, however, the overall WRFQ score showed a non-significant higher score among patients with ACRO and CD compared to patients with PRL and NFPA, indicating a trend towards better functioning at work. This seems at odds with the fewer paid jobs among patients with ACRO/CD. Even though it can be postulated that this is due to the smaller numbers, it can also be hypothesized that when patients with ACRO/CD are able to maintain their work, they appreciate their work more and therefore experience fewer work-related problems compared to patients with NFPA/PRL. We also found that the largest proportion of pituitary tumor patients were bothered at work by problems in the mental and social domains, which is in line with difficulties experienced by cancer survivors [38] and patients with rheumatoid arthritis [39].

A notable finding was the relatively low percentage of patients visiting an occupational physician (12%), despite the fact that quite a lot of patients (40%) in our study reported being bothered by health-related issues at work during the previous year. In the Netherlands, the occupational physician has the role of case manager to guide patients back to work. While patients in our cohort were in a chronic care setting, potentially explaining the minimal number of visits, this might be a potential target for future interventions.

### Strengths and weaknesses

A clear strength of this study is the large sample of participating patients, enabling comparison between various types of pituitary tumors. A recent study performed by van Lier et al. showed that the use of self-reported information on absenteeism and presenteeism was considered the best way to measure sick leave, quantity and quality of work [40], therefore supporting the results presented here.

The limitations of our study are mostly based on limitations of a cross-sectional cohort study. The non-longitudinal nature of the study leads to unanswered questions whether work disability in patients with pituitary tumors is due to the pituitary tumor or has a different nature, and on the interplay between productivity and quality of life. Another limitation is the single center setting of the study. Since the study took place in the Netherlands, which has relatively high work participation and high social security benefits, the generalizability to other countries might be an issue. The social security benefits in the Netherlands are not the same as but can be compared to systems in Scandinavia and Germany [41]. Due to the high social security benefits in the Netherlands, it could be that we observed higher rates of patients without a paid job compared to countries with lower benefits. This might also lead to more severe work problems in countries with lower social security benefits since these patients are unable to afford losing their jobs, and therefore work beyond their capacity, even though they are actually unable to keep it up.

An additional limitation is the distribution of the education level among participants in our study. Even though we invited all patients with a pituitary tumor, there was a high proportion of highly educated patients among our participants. This might decrease the generalizability of our study and the interpretation of our results, since in general, higher education reduces health-related working problems. Furthermore, the low amount of patients with a RCC patients is a limitation. However, after conducting a sensitivity analysis (excluding RCC from the analysis), no different effect was found.

Though quality of life and functioning are influenced by one’s ability to work and vice versa, these aspects are often overlooked in current care. A healthcare provider is not always aware of a patient’s employment status, and if so, it is generally difficult for the treating physician to help a patient to improve functioning at work. This emphasizes the relevance of our study and it remains important to realize that the impact of a pituitary tumor on work functioning is high. It is therefore necessary to increase awareness among all healthcare providers involved, including occupational physicians, and use targeted interventions in an effort to reduce work disability/prevent unemployment. Regarding interventions aimed at the problems perceived while being at work, these should focus on mental and social demands of the job in relation to the person’s capabilities.

## Conclusion

We have shown that work disability among patients with a pituitary tumor is substantial. Not only are they relatively often without a paid job, sick leave is considerable among those who work, and many patients encounter difficulties at work, mostly regarding the mental and social sphere. The determinants and difficulties at work found in this study could potentially be used for further research and we advise healthcare professionals to take these results into consideration in the clinical guidance of patients.

## Electronic supplementary material

Below is the link to the electronic supplementary material.

Supplementary material Figure 1 (DOCX 115 KB)

Supplementary material Figure 2 (DOCX 142 KB)

Supplementary material Table 1 (DOCX 17 KB)

Supplementary material Table 2 (DOCX 18 KB)

Supplementary material Table 3 (DOCX 29 KB)

## Abbreviations

NFPA: Non-functioning pituitary adenoma
ACRO: Acromegaly
CD: Cushing’s disease
PRL: Prolactinoma
RCC: Rathke’s cleft cyst
HRQoL: Health-related quality of life
SF-HLQ: Short Form-Health and Labour Questionnaire
WRFQ: Work Role Functioning Questionnaire 2.0
SF-36: Short form-36
EQ-5D: EuroQoL
LBNQ: Leiden Bother and Needs Questionnaire-Pituitary
SD: Standard deviation
IQR: Interquartile range
