# Supplementary material for: Work disability and its determinants in patients with pituitary tumor-related disease
Source: Pituitary. 2018 Oct 4;21(6):593–604. doi: 10.1007/s11102-018-0913-3 (PMC6244796; doi:10.1007/s11102-018-0913-3)
Supplement: Supplementary file 1 — Supplementary material Figure 1 (DOCX 115 KB) [file 11102_2018_913_MOESM1_ESM.docx]

**Supplementary figure 1.** Flow chart of employment status in patients with a pituitary tumor of working age
