# Supplementary material for: Work disability and its determinants in patients with pituitary tumor-related disease
Source: Pituitary. 2018 Oct 4;21(6):593–604. doi: 10.1007/s11102-018-0913-3 (PMC6244796; doi:10.1007/s11102-018-0913-3)
Supplement: Supplementary file 2 — Supplementary material Figure 2 (DOCX 142 KB) [file 11102_2018_913_MOESM2_ESM.docx]

**Supplementary figure 2.** Difficulties experienced at work among patients with a paid job (per tumor type and endocrine status)

**Flexibility demands**

**Mental and social demands**

**Physical demands**

**Work scheduling and output demands**

**Flexibility demands**

**Mental and social demands**

**Physical demands**

**Flexibility demands**

**Mental and social demands**

**Physical demands**

**Work scheduling and output demands**

**Work scheduling and output demands**

**Flexibility demands**

**Physical demands**

**Mental and social demands**

**Flexibility demands**

**Mental and social demands**

**Physical demands**

**Work scheduling and output demands**

**Flexibility demands**

**Mental and social demands**

**Physical demands**

**Work scheduling and output demands**

**Flexibility demands**

**Mental and social demands**

**Physical demands**

**Work scheduling and output demands**

**Flexibility demands**

**Mental and social demands**

**Physical demands**

**Work scheduling and output demands**
