# Supplementary material for: Work disability and its determinants in patients with pituitary tumor-related disease
Source: Pituitary. 2018 Oct 4;21(6):593–604. doi: 10.1007/s11102-018-0913-3 (PMC6244796; doi:10.1007/s11102-018-0913-3)
Supplement: Supplementary file 3 — Supplementary material Table 1 (DOCX 17 KB) [file 11102_2018_913_MOESM3_ESM.docx]

| **Supplementary table 1.** Treatment patterns of 241 patients with a pituitary tumor categorized per tumor type | | | | | | |
| --- | --- | --- | --- | --- | --- | --- |
|  | Total  (N=241) | NFA  (N=65) | ACRO  (N=41) | CD  (N=32) | PRL  (N=97) | RCC  (N=6) |
| Treatment, N (%) |  |  |  |  |  |  |
| **No treatment / discontinued medication** |  |  |  |  |  |  |
| No treatment | 9 (3.7) | 5 (7.7) | 0 (-) | 0 (-) | 3 (3.1) | 1 (16.7) |
| Discontinued medication | 27 (11.2) | 2 (3.1) | 0 (-) | 0 (-) | 24 (24.7) | 1 (16.7) |
| **Ongoing medication** |  |  |  |  |  |  |
| Medication only | 49 (20.3) | 1 (1.5) | 0 (-) | 0 (-) | 48 (49.5) | 0 (-) |
| Prior surgery | 18 (7.5) | 2 (3.1) | 10 (24.4) | 1 (3.1) | 5 (5.2) | 0 (-) |
| Prior surgery and radiotherapy | 8 (3.3) | 0 (-) | 6 (14.6) | 2 (6.2) | 0 (-) | 0 (-) |
| **Surgery** |  |  |  |  |  |  |
| Surgery only | 64 (26.6) | 30 (46.2) | 11 (26.8) | 19 (59.4) | 0 (-) | 4 (66.7) |
| Prior medication | 32 (13.3) | 6 (9.2) | 9 (22.0) | 5 (15.6) | 12 (12.4) | 0 (-) |
| **Radiotherapy** |  |  |  |  |  |  |
| Prior medication | 1 (0.4) | 0 (-) | 0 (-) | 0 (-) | 1 (1.0) | 0 (-) |
| Prior surgery | 25 (10.4) | 19 (29.2) | 1 (2.4) | 3 (9.3) | 2 (2.1) | 0 (-) |
| Prior medication and surgery | 8 (3.3) | 0 (-) | 4 (9.8) | 2 (6.3) | 2 (2.1) | 0 (-) |
| NFA (non-functioning pituitary adenoma), ACRO (acromegaly), CD (Cushing’s disease), PRL (prolactinoma), RCC (Rathke’s cleft cyst), N (number)  Due to rounding, not all percentages of the categorical variables add up to 100% | | | | | | |
