# Supplementary material for: Work disability and its determinants in patients with pituitary tumor-related disease
Source: Pituitary. 2018 Oct 4;21(6):593–604. doi: 10.1007/s11102-018-0913-3 (PMC6244796; doi:10.1007/s11102-018-0913-3)
Supplement: Supplementary file 4 — Supplementary material Table 2 (DOCX 18 KB) [file 11102_2018_913_MOESM4_ESM.docx]

| **Supplementary table 1.** Patient and work characteristics among 173 patients of working age with a pituitary tumor and a paid job, stratified per endocrine status | | | | | |
| --- | --- | --- | --- | --- | --- |
|  | **Total**  **(N=173)** | **No deficits**  **(N=94)** | **Hypopit**  **(N=50)** | **Panhypopit**  **(N=29)** | **p-value** |
| **SF-HLQ** | | | | | |
| Working hours/week, median (IQR) | 36.0  (24.0-40.0) | 32.0  (24.0-40.0) | 36.0  (27.0-40.0) | 38.0  (31.0-40.0) | .566† |
| Bothered by health-related problems during work, N (%) | 68 (39.3) | 30 (34.5) | 22 (46.8) | 16 (55.2) | .113† |
| Performance at work despite health-related problems, mean (SD) (scale 1-10)* | 6.8 (1.7) | 7.0 (1.5) | 6.4 (1.7) | 7.0 (2.0) | .579† |
| Absence from work during the past year due to health-related problems, N (%) | 70 (40.5) | 27 (28.7) | 15 (30.0) | 18 (62.1) | **.011**† |
| Days absent during previous year, median days (IQR) | 5.0  (4.0-28.0) | 5.0  (3.0-30.0) | 10.0  (3.0-55.0) | 8.5  (5.0-20.0) | **.017**† |
| **Medical consumption** | | | | | |
| Contact with occupational physician, N (%) | 21 (12.1) | 10 (10.6) | 9 (18.0) | 2 (6.8) | .279 |
| **WRFQ 2.0 (scale 0-100)** | | | | | |
| Work scheduling and output demands, mean (SD)* | 78.0 (28.7) | 82.9 (24.3) | 68.3 (34.4) | 78.3 (28.9) | **.016**† |
| Physical demands, mean (SD)* | 84.3 (27.1) | 88.5 (21.3) | 73.0 (35.5) | 88.6 (24.3) | **.006**† |
| Mental demands and social demands, mean (SD)* | 75.6 (31.2) | 82.9 (24.1) | 67.9 (34.5) | 77.8 (31.8) | **.019**† |
| Flexibility demands, mean (SD)* | 79.8 (29.1) | 84.3 (24.7) | 71.0 (34.6) | 79.1 (28.7) | **.042**† |
| Index score, mean (SD)* | 78.6 (28.1) | 83.9 (22.6) | 67.6 (34.3) | 79.2 (28.7) | **.003**† |
| (bold) p<0.05  * Higher scores indicate better performance at work  † corrected for age and gender  NFA (non-functioning pituitary adenoma), ACRO (acromegaly), CD (Cushing’s disease), PRL (prolactinoma), RCC (Rathke’s cleft cyst), N (number), SD (standard deviation), IQR (interquartile range), SF-HLQ (short form-health and labour questionnaire), WRFQ 2.0 (work role functioning questionnaire 2.0) | | | | | |
