# Supplementary material for: Work disability and its determinants in patients with pituitary tumor-related disease
Source: Pituitary. 2018 Oct 4;21(6):593–604. doi: 10.1007/s11102-018-0913-3 (PMC6244796; doi:10.1007/s11102-018-0913-3)
Supplement: Supplementary file 5 — Supplementary material Table 3 (DOCX 29 KB) [file 11102_2018_913_MOESM5_ESM.docx]

| **Supplementary table 2.** Percentages of perceived work-related difficulties at work per tumor type in patients with a pituitary tumor and a paid job | | | | | | | | | | | | |
| --- | --- | --- | --- | --- | --- | --- | --- | --- | --- | --- | --- | --- |
|  | **Total**  **(N=173)** | | **NFA**  **(N=50)** | | **ACRO**  **(N=25)** | | **CD**  **(N=17)** | | **PRL**  **(N=77)** | | **RCC**  **(N=4)** | |
| Difficulty with…. | Sometimes  (1-50%)  % of patients | Frequently  (50-100%)  % of patients | Sometimes  (1-50%)  % of patients | Frequently  (50-100%)  % of patients | Sometimes  (1-50%)  % of patients | Frequently  (50-100%)  % of patients | Sometimes  (1-50%)  % of patients | Frequently  (50-100%)  % of patients | Sometimes  (1-50%)  % of patients | Frequently  (50-100%)  % of patients | Sometimes  (1-50%)  % of patients | Frequently  (50-100%)  % of patients |
| **Work scheduling and output demands** |  |  |  |  |  |  |  |  |  |  |  |  |
| Get going easily at the beginning of the workday | 28.2 | 18.4 | 31.1 | 13.3 | 37.5 | 8.3 | 5.9 | 5.9 | 28.4 | 27.0 | 33.3 | 33.3 |
| Start on your job as soon as you arrived at work | 23.8 | 12.8 | 30.4 | 10.9 | 20.8 | 8.3 | 5.9 | 11.8 | 25.7 | 14.9 | 0.0 | 33.3 |
| Do your work without stopping to take extra breaks or rests | 30.6 | 16.6 | 30.2 | 16.3 | 33.3 | 12.5 | 31.3 | 12.5 | 29.6 | 18.3 | 33.3 | 33.3 |
| Stick to a routine or schedule | 20.7 | 14.6 | 17.0 | 19.1 | 27.3 | 4.5 | 29.4 | 11.8 | 20.3 | 13.5 | 0.0 | 50.0 |
| Work fast enough | 27.4 | 16.5 | **33.3** | **17.8** | 26.1 | 8.7 | 29.4 | 17.6 | 24.0 | 16.0 | 25.0 | 50.0 |
| Finish work on time | 30.7 | 16.6 | **37.8** | **17.8** | **41.7** | **8.3** | 13.3 | 13.3 | 26.7 | 17.3 | 25.0 | 50.0 |
| Do your work without making mistakes | 22.8 | 14.8 | 26.1 | 17.4 | 26.1 | 8.7 | 13.3 | 13.3 | 23.0 | 13.5 | 0.0 | 50.0 |
| Satisfy the people who judge your work | 18.8 | 15.6 | 20.9 | 23.3 | 25.0 | 12.5 | 0.0 | 12.5 | 20.5 | 11.0 | 0.0 | 50.0 |
| Feel a sense of accomplishment in your work | 25.7 | 18.0 | 29.8 | 17.0 | 16.7 | 20.8 | 17.6 | 11.8 | 28.0 | 17.3 | 25.0 | 50.0 |
| Feel you have done what you are capable of doing | 22.0 | 18.9 | 28.9 | 17.8 | 25.0 | 16.7 | 11.8 | 11.8 | 20.3 | 20.3 | 0.0 | 50.0 |
| **Physical demands** |  |  |  |  |  |  |  |  |  |  |  |  |
| Lift, carry, or move objects at work weighing more than 0 pounds | 17.8 | 16.1 | 20.0 | 16.7 | 10.0 | 10.0 | 25.0 | 8.3 | 18.5 | 18.5 | 0.0 | 50.0 |
| Sit, stand, or stay in one position for longer than 5 min while working | 17.8 | 14.0 | 23.3 | 11.6 | 20.0 | 16.0 | 0.0 | 20.0 | 18.3 | 12.7 | 0.0 | 33.3 |
| Repeat the same motions over and over again while working | 16.9 | 12.5 | 15.4 | 15.4 | 25.0 | 10.0 | 23.1 | 7.7 | 13.3 | 11.7 | 25.0 | 25.0 |
| Bend, twist, or reach while working | 16.1 | 12.6 | 12.5 | 12.5 | 21.7 | 4.3 | 14.3 | 14.3 | 17.5 | 14.3 | 0.0 | 33.3 |
| Use hand-held tools or equipment (for example, a phone, pen, keyboard, computer mouse, drill, hairdryer or sander) | 11.6 | 10.3 | 7.0 | 11.6 | 8.7 | 4.3 | 20.0 | 6.7 | 14.1 | 11.3 | 0.0 | 33.3 |
| **Mental and social demands** |  |  |  |  |  |  |  |  |  |  |  |  |
| Keep your mind on your work | 34.7 | 15.0 | 27.7 | 19.1 | **37.5** | **12.5** | 23.5 | 11.8 | **41.3** | **12.0** | 25.0 | 50.0 |
| Do work carefully | 26.7 | 13.3 | 23.4 | 19.1 | 26.1 | 13.0 | 18.8 | 12.5 | 32.0 | 8.0 | 0.0 | 50.0 |
| Concentrate on your work | **38.3** | **14.4** | **36.2** | **19.1** | 29.2 | 8.3 | 29.4 | 11.8 | **44.0** | **12.0** | 50.0 | 50.0 |
| Work without losing your train of thought | **39.4** | **15.8** | **36.2** | **21.3** | 29.2 | 8.3 | 46.7 | 6.7 | **44.0** | **14.7** | 25.0 | 50.0 |
| Easily read or use your eyes when working | **32.3** | **18.6** | 23.4 | 23.4 | 39.1 | 4.3 | 26.7 | 13.3 | **37.0** | **20.5** | 33.3 | 33.3 |
| Speak with people in-person, in meetings or on the phone | 14.0 | 13.4 | 21.3 | 14.9 | 8.0 | 12.0 | 6.7 | 6.7 | 13.5 | 13.5 | 0.0 | 33.3 |
| Control your temper around people when working | 13.8 | 12.5 | 11.4 | 15.9 | 13.0 | 8.7 | 6.3 | 6.3 | 17.6 | 12.2 | 0.0 | 33.3 |
| **Flexibility demands** |  |  |  |  |  |  |  |  |  |  |  |  |
| Set priorities in my work | 23.2 | 14.0 | 25.5 | 17.0 | 20.8 | 8.3 | 18.8 | 12.5 | 24.3 | 13.5 | 0.0 | 33.3 |
| Handle changes in my work | 24.7 | 16.0 | **33.3** | **17.8** | 20.8 | 8.3 | 18.8 | 12.5 | 23.0 | 17.6 | 0.0 | 33.3 |
| Process incoming information, for example e-mails, in time | 29.1 | 12.7 | **32.6** | **19.6** | 29.2 | 4.2 | 28.6 | 7.1 | 28.2 | 11.3 | 0.0 | 33.3 |
| Perform multiple tasks at the same time | 28.7 | 17.7 | **27.7** | **25.5** | 24.0 | 12.0 | 25.0 | 18.8 | 31.5 | 13.7 | 33.3 | 33.3 |
| Be proactive, show initiative in my work | 16.4 | 12.1 | 17.4 | 17.4 | 16.0 | 12.0 | 5.9 | 11.8 | 18.9 | 8.1 | 0.0 | 33.3 |
| (bold) ≥50% of patients experience problems with item at work (only considering NFA, ACRO, CD, PRL based on the low number of patients with a RCC)  NFA (non-functioning adenoma), ACRO (acromegaly), CD (Cushing’s disease), PRL (prolactinoma), RCC (Rathke’s cleft cyst)  The 5-point rating scale is categorized into three categories: 1) completely not (0%), 2) sometimes (“some of the time” and “half of the time (50%)”) and 3) frequently (“most of the time” and “all the time” (100%)) | | | | | | | | | | | | |
